# Improved Prediction of Non-methylated Islands in Vertebrates Highlights Different Characteristic Sequence Patterns

**DOI:** 10.1371/journal.pcbi.1005249

**Published:** 2016-12-16

**Authors:** Matthew Huska, Martin Vingron

**Affiliations:** Computational Molecular Biology, Max Planck Institute for Molecular Genetics, Berlin, Germany; Ottawa University, CANADA

## Abstract

Non-methylated islands (NMIs) of DNA are genomic regions that are important for gene regulation and development. A recent study of genome-wide non-methylation data in vertebrates by Long et al. (eLife 2013;2:e00348) has shown that many experimentally identified non-methylated regions do not overlap with classically defined CpG islands which are computationally predicted using simple DNA sequence features. This is especially true in cold-blooded vertebrates such as *Danio rerio* (zebrafish). In order to investigate how predictive DNA sequence is of a region’s methylation status, we applied a supervised learning approach using a spectrum kernel support vector machine, to see if a more complex model and supervised learning can be used to improve non-methylated island prediction and to understand the sequence properties of these regions. We demonstrate that DNA sequence is highly predictive of methylation status, and that in contrast to existing CpG island prediction methods our method is able to provide more useful predictions of NMIs genome-wide in all vertebrate organisms that were studied. Our results also show that in cold-blooded vertebrates (*Anolis carolinensis*, *Xenopus tropicalis* and *Danio rerio*) where genome-wide classical CpG island predictions consist primarily of false positives, longer primarily AT-rich DNA sequence features are able to identify these regions much more accurately.

## Author Summary

DNA methylation is one of the most heavily studied epigenetic modifications, with active research into the topic taking place for over 30 years. Over that time a relationship between non-methylated stretches of genomic DNA and the regulation of nearby genes has been identified, and it was noted that approximately half of all human genes have an unexpectedly high number of unmethylated CpG dinucleotides in their promoters. Nevertheless, until relatively recently we haven’t been able to directly measure methylation gnome-wide, leading to the widespread use of CpG-rich regions (or CpG Islands) as a proxy for unmethylated genomic regions. Fortunately, recently developed experiments finally allow us to look at DNA methylation genome-wide, and have shown that CpG islands do not overlap true non-methylated islands to the extent we would have hoped, especially in cold-blooded vertebrates. We show that by using these experimentally determined non-methylated regions as training data, we are able to improve the prediction of non-methylated regions genome-wide in comparison to classical CpG island prediction methods, in both warm-blooded and cold-blooded vertebrates. Additionally, we show that while CpG content is an important predictor of methylation status in warm-blooded vertebrates, in cold-blooded vertebrates higher predictive accuracy can be achieved using longer stretches of relatively AT-rich DNA sequence.

## Introduction

DNA methylation is known to play an important role in vertebrate gene regulation [1, 2]. Most of the human genome is usually methylated, however over 30 years ago a relatively small number of non-methylated regions were identified using methylation-sensitive restriction enzymes [3]. These non-methylated regions were found to have a higher than expected number of CpG dinucleotides when compared to the rest of the genome, and it was suggested that this is because methylated CpGs are more likely to be mutated to CpAs or TpGs than non-methylated CpGs, leading to the reduction of CpG dinucleotides in most of the genome [4, 5]. Due to the increasing availability of genomic sequence data this sequence-based definition of non-methylated regions became popular, and they have come to be referred to as CpG islands. One of the first sequence-based definitions of CpG islands was proposed by Gardiner-Garden at al. [6], which defines CpG islands as regions of the genome that have a length of >200bp, GC content >50%, and a ratio of observed CpGs to expected CpGs (or CpG Ratio) >0.6. A variant of this method is still used to provide an annotation of CpG islands in the popular UCSC Genome Browser [7], and for many years it has been used as a proxy for non-methylated regions of the genome.

CpG ratios are also used in vertebrates to classify genes into those with high CpG promoters and low CpG promoters, due to the observation that there is a clear bimodal pattern in the CpG ratios of human promoters [8, 9]. This bimodality is also observed in several other vertebrates including humans, chicken, frog and zebrafish [10]. However, the overall percentage of promoters that overlap a CpG island was later shown to be much lower in cold-blooded vertebrates (<20%) when compared to warm-blooded vertebrates (>40%) [11]. This suggested that very few promoters are unmethylated in cold-blooded vertebrates, or that the role of CpG-rich regions may differ between these two groups of organisms.

Later, tissue-specific methylated regions were identified and found to be correlated with tissue-specific gene expression. One earlier study [12] showed that out of 150 differentially methylated regions that were studied, 100 of the regions overlapped with predicted CpG islands, many of which were shown to be methylated in most tissues. More recent papers have reported similar results on a genome-wide scale [13, 14]. This dynamic methylation, even at CpG islands, made it clear that some disagreement between true unmethylated regions and CpG island predictions should be expected since CpG island predictions were not tissue-specific. Additionally, fish CpG islands were found to be GC-poor though thought to be still have a high CpG observed/expected ratio [15], and a study of CpG islands in mouse and human showed that the GC content of islands in the two organisms differs [16]. These findings made it clear that a single model of CpG islands for all of these species would not be appropriate, and at the very least the cutoffs for calling a region a CpG island would need to be adapted when applying the prediction to other organisms.

Fortunately, experimental methods have now been developed that are able to identify methylated or non-methylated regions (also called non-methylated islands, or NMIs) in a given cell line or tissue genome-wide. These methods include bisulfite sequencing [17, 18] as well as the Bio-CAP method [19], the latter of which is specifically for identifying regions that are unmethylated. Recently, Bio-CAP was used to determine the location of NMIs in several vertebrates, including both warm-blooded (human, mouse, chicken and platypus) and cold-blooded (lizard, frog and zebrafish) vertebrates, in multiple tissues including testes and liver [20]. It was noted that the number of non-methylated regions that overlap with predicted CpG islands was in most cases quite low (e.g. ≈ 20% overlap in Zebrafish), leading the authors of that study to conclude that the computational methods that are commonly used to identify CpG islands are not able to accurately identify NMIs in vertebrate genomes. The same study [20] also showed that in contrast to the low percentage suggested by CpG island predictions, over 50% of promoters in all vertebrates actually do have non-methylated regions at their TSSs, but that existing CpG island prediction methods are simply not able to identify them in cold-blooded vertebrates.

We investigated whether it is possible to use computational methods to predict the location of NMIs in the genomes of six vertebrates using only the DNA sequence itself. The method we used differs from methods that are used for the computational prediction of CpG islands in two ways: first, we use newly available experimentally determined non-methylated island regions as training data and apply a supervised learning approach in order to learn from these regions. By training our model separately on each organism and tissue, it is possible to predict tissue-specific NMIs from genomic sequence, despite the fact that genomic sequence is identical between tissues, as well as adapt to different organism specific mechanisms and genome-wide properties such as differing background GC content. This was not possible when classical CpG island prediction methods were developed because experimental methods that could identify non-methylated regions in a large scale did not yet exist, so these methods had to rely on unsupervised learning approaches which do not leverage experimentally determined NMI regions. Second, as a classifier we use a string kernel support vector machine (SVM) which allows us to easily use longer subsequences of DNA as features, rather than looking only at dinucleotide frequencies. Support vector machines have been used successfully to identify other types of regulatory sequences [21, 22] as well as in the prediction of DNA methylation itself [23–25]. However, with the exception of a cursory analysis of NMIs [26], this is the first genome-wide analysis of the predictive power of DNA sequence across such a wide range of vertebrate species using uniformly generated and analyzed data.

Our study reveals that there are strongly predictive sequence features that differentiate NMIs from the rest of the genome in all six organisms that we studied (area under the ROC curve of a genome-wide classifier 0.91–0.99). In contrast to predicting NMIs using the regions’ CpG ratios only, the SVM is able to maintain a much lower FDR for all organisms, especially cold-blooded vertebrates (lizard, frog and zebrafish), where CpG ratio-based predictions contain an overwhelming number of false positives. Additionally, we show that longer sequence features (approximately 6 bp subsequences or longer) are required to predict NMIs in cold-blooded vertebrates, while shorter subsequences (2–4 bp) are sufficient in warm-blooded vertebrates. The most highly weighted sequence features are shown to contain a high number of C/G nucleotides in warm-blooded vertebrates, and a low number of C/G nucleotides in cold-blooded vertebrates.

Clearly, when NMIs are known for a given biological sample it is not necessary to predict them. Therefore, besides the lessons we have learned from constructing the classifier in the first place, we also assessed the ability of our classifier to be trained on one organism and to predict the NMIs in another. We show that cross-species prediction performs well across warm-blooded vertebrates, where the classifier is essentially an improved CpG island detector. However, in cold-blooded organisms where we have shown that the sequence features required to identify NMIs are more complex, this cross-species prediction is more difficult. It performs better in lizard than in frog and zebrafish, but none of the cold-blooded vertebrates have their NMIs predicted with the same level of accuracy as the NMIs in warm-blooded vertebrates.

## Results

### DNA Sequence Is Highly Predictive of Non-methylated Islands

To investigate how informative DNA sequence is in determining whether or not a given genomic region is a non-methylated island, we used a subset of each organism’s chromosomes to train an SVM to differentiate between known NMI sequences from testes and the rest of the genome (see Methods section for details). This classifier was then used to predict NMIs in the rest of the genome and its performance was evaluated. This process was carried out separately for each organism, which allowed us to identify differences in predictive performance and important sequence features between the six species. As a baseline, the performance of the classifier was compared to the observed versus expected CpG ratio of the sequence, as well as to two existing CpG island predictions: UCSC’s predictions based on the Gardiner-Garden and Frommer method [6] and a more recent hidden Markov model-based method [27]. We compare the performance of the methods using ROC curves and Precision-Recall curves in order to understand the performance of the classifiers when classifying a given random genomic window as an NMI or not, as well as whether the classifiers can annotate the entire genome while controlling the number of false positives.

**Fig 1:**
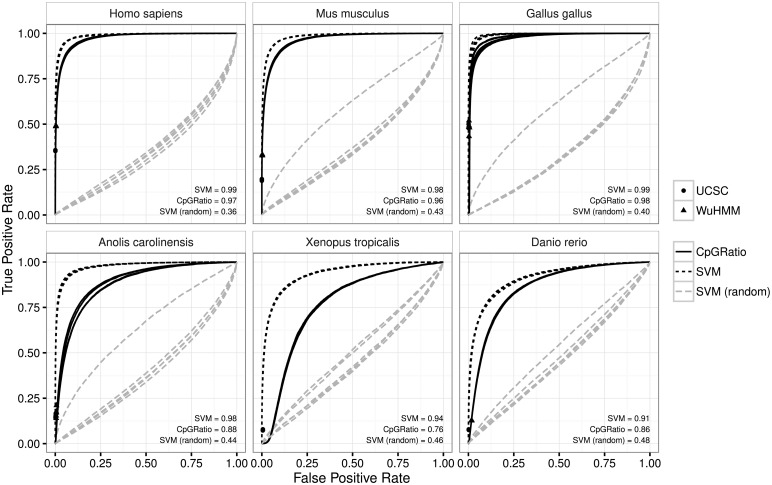
Receiver Operating Characteristic curves show that the DNA sequence is highly predictive of non-methylated regions, and our SVM method achieves higher AUROC than other methods when predicting these regions. A receiver operating characteristic curve for four different classifiers: SVM (our spectrum kernel SVM), CpG ratio (the ratio of observed versus expected CpG dinucleotides), UCSC CpG island predictions (a variant of the observed versus expected method with additional constraints), and Wu HMM (an HMM-based CpG island prediction method), as well as an SVM trained on sequences with randomly shuffled labels, “SVM (random)”. The UCSC and Wu HMM methods are shown as points rather than curves, because they only provide a set of genomic windows rather than scores for the whole genome, essentially the same as choosing a single cutoff score for the other methods. The prediction was run five times with different random splits of training and test data, therefore five lines or points are shown for each method. The performance is very stable between runs, with the lines for each run almost perfectly overlapping. The average area under the curve across all 5 random splits is indicated in each panel.

Our ROC curves show that our classifier was able to identify NMIs based on sequence alone with high performance (see Fig 1 and Table 1). A simple CpG ratio is also quite predictive in humans, mice, and chickens (AUROC 0.96–0.98), though our SVM performs better in all cases (AUROC 0.98–0.99). In cold-blooded vertebrates our SVM is able to improve NMI prediction even more markedly over the CpG ratio (SVM AUROC 0.91–0.98 versus CpG ratio AUROC 0.76–0.88). The UCSC and Wu HMM methods are both very conservative with their predictions. While they both maintain low false positive rates, they also have very low average true positive rates of between 7% and 49%. The two methods also achieved higher true positive rates in warm-blooded vertebrates than cold-blooded vertebrates, with an average of 3 times more true positives being identified in warm-blooded vertebrates than in cold-blooded vertebrates.

**Table 1:** Our SVM achieves higher AUROC and AUPRC than CpG ratio-based predictions for every organism. The table shows the average AUROC and AUPRC of whole genome NMI prediction across 5 random splits of parameter tuning, training and test data. The performance of an SVM trained on sequences with randomly shuffled labels is included as “SVM (random)”.

	AUROC			AUPRC		
organism	CpGRatio	SVM	SVM (random)	CpGRatio	SVM	SVM (random)
Homo sapiens	0.974	0.988	0.356	0.584	0.820	0.013
Mus musculus	0.964	0.983	0.425	0.611	0.774	0.024
Gallus gallus	0.975	0.993	0.397	0.433	0.849	0.019
Anolis carolinensis	0.883	0.977	0.443	0.105	0.664	0.014
Xenopus tropicalis	0.764	0.937	0.462	0.029	0.400	0.012
Danio rerio	0.857	0.913	0.477	0.115	0.416	0.023

### Support Vector Machine Is Uniquely Able to Predict NMIs Genome-Wide in All Six Vertebrates

**Fig 2:**
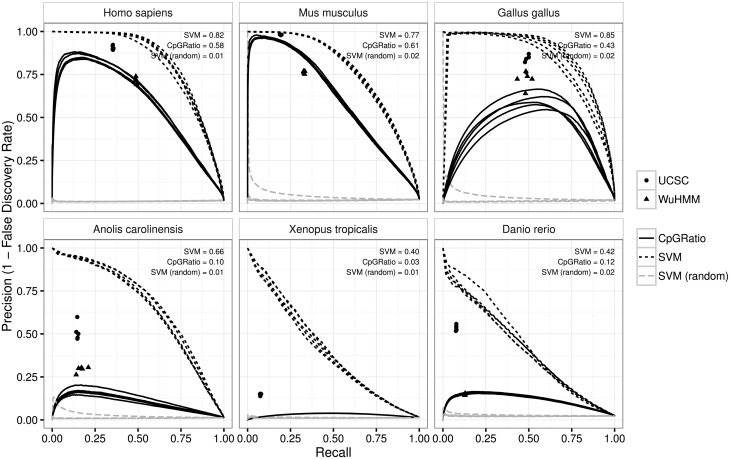
Precision-Recall curves show that the SVM is better able to provide genome-wide NMI predictions while controlling for false positives. These curves plot the relationship between the fraction of correctly identified regions (precision) versus the fraction of all NMIs that are identified (recall). The SVM method performs better than all other methods, with a higher AUPRC in every organism. The CpG ratio method performs very poorly cold-blooded vertebrates (lizard, frog and zebrafish), and in the case of frog never achieves a precision higher than 0.1 regardless of recall. The average area under the curve across all 5 random splits of the genome into parameter tuning, training and test sets is indicated in each panel. The performance of an SVM classifier trained on sequences with randomly shuffled labels, “SVM (random)”, is shown in grey.

Our Precision-Recall curves (PRCs) more clearly show that the SVM outperforms the other methods, and despite the fact that the ROC curves for the SVM and CpG Ratio looked quite similar the PRC shows that the SVM is much better at controlling false positives genome-wide (see Fig 2). In the case of all cold-blooded vertebrates (lizard, frog and zebrafish) the CpG ratio-based predictions consist almost exclusively of false positives, regardless of the scoring cutoff that is used. In contrast, the SVM-based method is still able to control the amount of false positives, though at an admittedly low recall. The UCSC and Wu HMM methods both have high precision in warm-blooded vertebrates (>0.71 in all cases), but their precision drops in cold-blooded vertebrates. The UCSC method still manages an average of 0.52 precision in lizard and zebrafish, but this decreases to 0.15 in frog. The Wu HMM method has lower performance, with an average precision of 0.3 in lizard and 0.15 in zebrafish. The Wu HMM software was not able to produce a prediction for frog (the software crashed repeatedly). While some of the false positives which lead to low precision for many of the predictions could in fact be false negatives from the Bio-CAP method, we have no way of knowing if this is the case from the Bio-CAP data alone.

### Longer CpG-Poor Sequence Features Are Required for Accurate NMI Identification in Cold-Blooded Vertebrates

**Fig 3:**
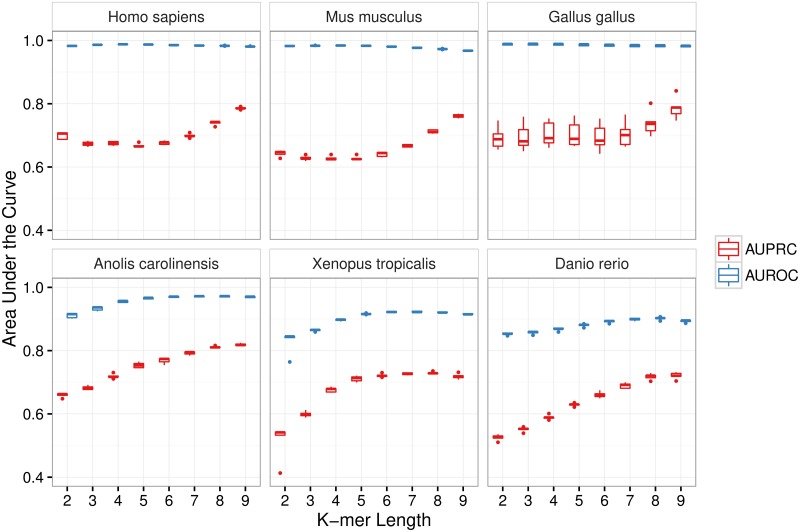
Longer DNA subsequences are required to accurately identify NMIs in cold-blooded vertebrates. While the frequency of di- and tri-nucleotides is already highly predictive of NMI status in warm-blooded vertebrates (AUROC >0.97), the frequencies of k-mers of length 6 or more are required for accurate prediction of NMIs in cold-blooded vertebrates. Box plots show the prediction performance on the parameter tuning set across 5 runs of 5-fold cross validation. The data sets for all organisms consist of 30,000 750bp windows with a 5:1 ratio of non-NMI windows to NMI windows. This fixed number of windows and fixed class imbalance means that both the AUROC (a) and AUPRC (b) can be compared across organisms.

**Fig 4:**
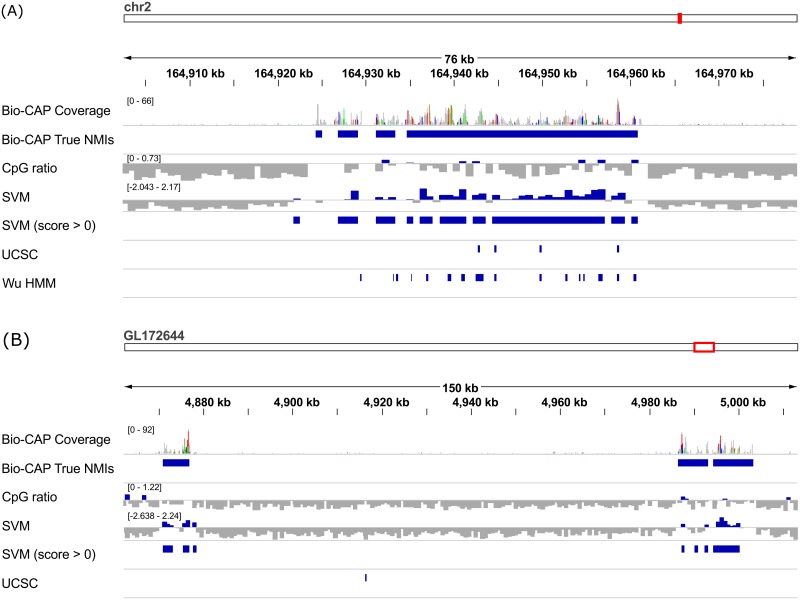
Example regions in frog and lizard showing the improvement of using longer k-mers rather than CpG-based measures Two example regions from (A) Anolis carolinensis and (B) Xenopus tropicalis. In both cases there are NMIs that contain stretches of relatively low CpG content, which are either poorly predicted (in lizard) or not predicted at all (in frog) using CpG ratios, UCSC CpG island predictions or the Wu HMM method. Nevertheless they are quite accurately predicted using the SVM-based method that uses longer k-mers as features.

We next sought to investigate why CpG island prediction methods based on CpG ratios perform so poorly at predicting NMIs in cold-blooded vertebrates in comparison to their higher predictive accuracy in warm-blooded vertebrates. Additionally, we wanted to understand why CpG ratios perform so poorly in cold-blooded vertebrates while our classifier is better able to control false positives. To address these questions we looked into the performance of our classifier on the parameter tuning subset of the data, which contained the same number of windows for all organisms (30,000) and a fixed ratio of non-NMI windows to NMI windows (5:1). For all datasets we calculated the AUROC and AUPRC when using k-mers of increasing length (see Methods section) as features (see Fig 3). The results show that short k-mers are already very predictive of NMI status in warm-blooded vertebrates (AUROC >0.97), but that longer k-mers are required for reasonable performance in cold-blooded vertebrates, especially frogs and zebrafish. Using longer k-mers that led to a high AUROC in all organisms we observed that the 20 highest scoring k-mers were almost completely devoid of A/T nucleotides in warm-blooded vertebrates, while nearly every k-mer in cold-blooded vertebrates contained an A or T nucleotide (see Table 2). Two example regions in frog and lizard are shown in Fig 4, where in some windows overlapping an NMI the CpG ratio is low, the UCSC and Wu HMM methods both perform poorly, but the SVM classifier is still able to correctly identify the majority of the NMI region.

**Table 2:** The Top 20 k-mers according to a 6-mer kernel SVM in all six organisms. The highest ranked k-mers contribute the most to classifying a region as a non-methylated island. K-mers containing more than one A or T nucleotide are in bold. A and T nucleotides are almost completely absent from high scoring 6-mers in warm-blooded organisms, while nearly every high scoring 6-mer contains an A or T in cold-blooded organisms.

	Homo sapiens	Mus musculus	Gallus gallus	Anolis carolinensis	Xenopus tropicalis	Danio rerio
1	CCCCGC	GGGCGG	GGGGGG	**TGTGTG**	**ATCTAT**	GCGCGC
2	CCGCCC	CCGCCC	CCCCCC	**AAAAAA**	**ATAGAT**	CGCGCG
3	GCGGGG	CGCCCC	**AAAAAA**	**TCTCTC**	**GATAGA**	**ACACAC**
4	GGGGCG	GGGGCG	**TTTTTT**	**GTGTGT**	**TCTATC**	**GTGTGT**
5	CCCGCC	CCCGCC	GCGGGG	CCCCCC	**ACACAC**	**TAACGT**
6	CGCCCC	GCGCGC	CCCCGC	**AGAGAG**	**GTGTGT**	**ACGTTA**
7	GGCGGG	CCCCGC	GCAGCG	**GAGAGA**	**ATATAT**	**AACGTT**
8	GGGCGG	**AAAAAA**	GCCCCG	**CTCTCT**	**TAGATA**	**TTTAAA**
9	GCGCGC	GCGGGG	CGCTGC	**CACACA**	**TATCTA**	**CACACA**
10	CGCGCC	GGCGGG	CAGCGC	GGGGGG	**CACACA**	**TTAAAT**
11	GGCGCG	CGCGCG	CGCAGC	**TTTTTT**	**TGTGTG**	**TCTCTC**
12	GCGCGG	GCCCCG	CGGGGC	**ACACAC**	**AGATAG**	**CTCTCT**
13	CCGCGG	CTCCGC	GCTGCG	**ATATAT**	**CTATCT**	**TGTGTG**
14	CCGCGC	CCCCCC	GCGCTG	**CCTTCC**	**TATATA**	CGCGCT
15	CGCGGG	CTGCGC	GGCAGC	**GGAAGG**	**AAAAAA**	ACGCGC
16	CCCGGG	CCCGGG	GCTCCG	**GAGGAG**	**TTTTTT**	**GAGAGA**
17	CCCGCG	GCGGAG	CCCGGC	GCGCGC	**AGCAGC**	AGCGCG
18	CTCCCG	CGGGGC	GGGCGG	**CCTCCT**	**GCTGCT**	**TATCTA**
19	GCGGAG	GCTGCG	GCCGGG	**GGAGGA**	**GCAGCA**	**AGAGAG**
20	GCCCCG	GGGCGC	CAGCCC	**AGGAGG**	**TGCTGC**	**ATTTAA**

### Cross-Species Prediction

We also investigated how well a classifier trained in one organism can predict NMIs in another organism. This is particularly useful because it would potentially allow us to improve NMI annotation in species whose genomic sequence is known but genome-wide methylation experiments such as Bio-CAP or whole-genome bisulfite sequencing have not yet been performed, which is the majority of species. In order to make it easier to compare the results across organisms, we fixed the SVM parameters and used a k-mer length of 6 for all organisms. This way we can attribute any differences that we observe to the differences between organisms rather than differences in parameters. Additionally, after observing the clear grouping of samples into cold and warm-blooded vertebrates in the previous results, we decided to add two more training sets to the analysis: a set of sequences sampled from all warm-blooded vertebrate species (human, mouse and chicken), and a set of sequences from all cold-blooded species (lizard, frog and zebrafish).

As shown in Table 3, the best predictions (AUPRC) are always achieved when training and testing on the same species. The pooled samples which include the test organism consistently achieve the second best performance. The one exception to this was in chicken, where the prediction based on pooled warm-blooded sequences was slightly better than the predictions using chicken sequences alone, though this difference is very small (0.003 AUPRC). In general the warm-blooded vertebrates are all fairly well predicted by other warm-blooded vertebrates as well as by lizard. A classifier trained on frog and zebrafish sequences performed relatively poorly when trying to predict NMIs in any of the warm-blooded vertebrates, though pooled cold-blooded sequences managed to achieve a high AUPRC in chicken (0.792). Cross-species prediction of NMIs in cold-blooded species was overall very difficult. The only partial exception to this is the lizard, whose NMIs were predicted modestly well (> 0.3 AUPRC) by classifiers trained in any organism or set of organisms, with the exception of frog and zebrafish. These results also show that best cross-species predictor for warm-blooded organisms is always the organism that has the most recent common ancestor: mouse and human are more closely related than chicken, and also achieve the highest cross-species predictive performance. Additionally, the predictor based on pooled warm-blooded vertebrate species would be useful for predicting NMIs in other warm-blooded vertebrates as well as lizard, a species whose last common ancestor with the three warm-blooded organisms was roughly 300 million years ago. This is not the case in the remaining cold-blooded vertebrates, where there is no trend of better cross-species prediction when training on more evolutionarily close organisms.

**Table 3:** Results of training a classifier in one species and predicting in another. Rows are the organisms which the classifier was trained on, and columns are the organisms which the classifier was tested on. The last two rows show the performance when training on a set of combined sequences from human, mouse and chicken (All warm-blooded), or lizard, frog and zebrafish (All-coldblooded). Each value is the mean AUPRC across 5 separate training sets and test sets (see Methods for details). The tissue in all cases was testes.

	Homo sapiens	Mus musculus	Gallus gallus	Anolis carolinensis	Xenopus tropicalis	Danio rerio
Homo sapiens	0.815	0.709	0.829	0.339	0.047	0.107
Mus musculus	0.747	0.752	0.831	0.332	0.052	0.096
Gallus gallus	0.685	0.635	0.835	0.330	0.088	0.092
Anolis carolinensis	0.702	0.548	0.770	0.658	0.059	0.088
Xenopus tropicalis	0.392	0.351	0.598	0.254	0.410	0.086
Danio rerio	0.497	0.349	0.665	0.164	0.039	0.400
All warm-blooded	0.781	0.726	0.838	0.353	0.059	0.098
All cold-blooded	0.664	0.547	0.792	0.564	0.183	0.217
CpG Ratio	0.584	0.611	0.433	0.105	0.029	0.115

## Discussion

Our results demonstrate that highly informative DNA sequence features are contained within experimentally determined non-methylated regions of DNA in vertebrates, including cold-blooded vertebrates. Using known non-methylated regions of the genome to both train and evaluate our methods, we were able to show that DNA sequence can be used as a predictor for non-methylated regions in all six vertebrates that were examined. This was not entirely expected because existing sequence-based CpG island predictions have a low overlap with true non-methylated regions, suggesting that DNA sequence might not be highly predictive of non-methylated regions [20]. The high performance of our classifier in all organisms proves that despite the low overlap with existing predictions, the use of a more complex model with longer subsequences as features and a supervised learning approach demonstrates that there are DNA features in all vertebrates that are predictive of NMI regions.

Secondly, these informative DNA features can be used to predict the location of non-methylated islands genome-wide better than existing CpG island prediction methods. Given the fact that NMIs only make up 2–4% of the genome, only a classifier that can control false positives will be useful for predicting non-methylated regions genome-wide. The precision-recall curves show that for all organisms we are able to predict NMIs better than all existing methods, and that for cold-blooded vertebrates we are uniquely able to identify a modest number of non-methylated regions while maintaining a low false discovery rate.

Additionally, we have shown that NMI predictions made by training a classifier in one species and predicting in another can be highly accurate when performed between warm-blooded species and to a lesser extent lizard, while cross-species prediction in zebrafish and frog proved to be very difficult. One explanation for the differences we are observing in cross-species prediction accuracy is that NMI regions in all six vertebrates are identified based on two factors: first, simple CpG-richness, and second, more complex sequence features (potentially including transcription factor binding sites) that are species and/or tissue-specific. These two factors have a different level of importance in each organism. In warm-blooded vertebrates, the first factor is far more important than the second, while in cold-blooded vertebrates the second is more important than the first. This is why a simple classifier using dinucleotide frequencies works so well in warm-blooded vertebrates, and why this simple classifier is easily transferable to other warm-blooded vertebrates. In contrast, in zebrafish and frog CpG content still contributes to the prediction of NMIs, but the contribution of more complex sequence features is more important. These complex sequence features are species-specific, and therefore do not transfer well between species, resulting in poor cross-species prediction accuracy in zebrafish and frog. Lastly, the importance of the two factors in lizard is more balanced. CpG content is more important than in other cold-blooded vertebrates, but complex sequence features are more important than in warm-blooded vertebrates. This explains why we observe fairly good prediction of NMIs in lizard after training in warm-blooded vertebrates, which are identifying the simple CpG richness that is somewhat important in lizard NMIs. On the other hand, the classifiers trained in other cold-blooded vertebrates have learned to identify complex species-specific sequence features and place less importance on CpG richness, and therefore perform poorly at predicting lizard NMIs.

The importance of longer, more complex sequence features, especially in frog and zebrafish, suggests that different mechanisms for the establishment and maintenance of non-methylated regions may be dominant in these organisms. To investigate this, the k-mers that contribute the most to the classification of NMIs in each organism were compared to all known transcription factor binding motifs in the JASPAR 2016 vertebrates database [28]. In warm-blooded vertebrates as well as lizard, the resulting enriched transcription factor motifs included a number of zinc finger proteins and other DNA binding proteins with GC-rich binding motifs (e.g. SP1, SP2, and E2F family proteins), as had been previously observed [2]. In the remaining cold-blooded vertebrates there were essentially no enriched motifs within NMI regions (see data in S2 Table).

The important k-mers identified by the SVM did show clear differences between warm-blooded and cold-blooded organisms though, with the highest scoring k-mers containing almost no A/T nucleotides in warm-blooded organisms, and nearly every high scoring k-mer containing an A/T nucleotide in cold-blooded organisms (see Table 3). This disagreement between the importance of the k-mers, which suggests a role for sequence-specific transcription factors, and the lack of known JASPAR motifs could suggest that the factors involved in establishing and maintaining NMIs in cold-blooded organisms may have changed along with composition of cold-blooded NMI sequences themselves. And because of this our databases do not adequately reflect those transcription factor binding sites in cold-blooded vertebrates.

Overall, these findings are complemented by those presented in a new paper which was published during the review process of this article [29]. In that paper the authors experimentally evaluated whether stretches of genomic sequence containing NMIs from one organism can also evade methylation when placed into another organism. Their study demonstrated that transplanting a segment of genomic DNA from human into mouse, or from mouse into zebrafish, resulted in the majority of NMIs within these regions remaining unmethylated. In particular, these findings show that the fish is capable of protecting mouse CpG islands from being methylated. Nevertheless, the typical fish NMI sequence has evolved in the direction of the sequence patterns that we identified in our analysis. This again raises the point that there should be a particular functional pressure that has led it to do so, for example transcription factors whose motifs are not currently known in cold-blooded vertebrates.

## Methods

### Data

The locations of experimentally determined NMIs in the testes of six vertebrates were taken from a recent paper [20], which we used as a gold standard set of true NMIs. These NMIs were identified using the Bio-CAP method [19], which provides a genome-wide estimate of the proportion of unmethylated CpG dinucleotides. In comparison to another popular method for measuring methylation levels, whole genome bisulfite sequencing, Bio-CAP relies on affinity purification of non-methylated CpGs with a zinc finger CxxC domain, has lower resolution and in at least one set of experiments identifies a substantially lower number of non-methylated regions (approximately 41,000 human NMIs with Bio-CAP [20] compared to 51,572 human NMIs with bisulfite sequencing [14]), suggesting that Bio-CAP results may contain substantial number of false negative regions. Despite these potential shortcomings, this data set was selected because it is the only one available that covers a sufficiently wide range of vertebrate species in matching tissues. The six vertebrates that we analyzed, along with their UCSC genome version, were *Homo sapiens* (hg19), *Mus musculus* (mm9), *Gallus gallus* (galGal3), *Anolis carolinensis* (anoCar2), *Xenopus tropicalis* (xenTro3), and *Danio rerio* (danRer7). Reference genomes were downloaded from UCSC to match the versions used in [20]. We omitted the platypus because its genome assembly is highly fragmented, consisting of over 200 thousand separate sequence fragments, and only approximately 17% of the organism’s genome could be mapped to a chromosome and ordered [30]. Experimentally determined NMI locations from liver tissue in all six organisms were also analyzed and the resulting trends were the same as those for testes, so we omitted those results from the paper for the sake of conciseness.

### Existing CpG Island Predictions

CpG island predictions from UCSC’s Genome Browser were downloaded from the UCSC FTP site. These predictions are based on a variant of the original Garden-Gardiner and Frommer method [6], with a number of modifications: the length of the region must be ≥ 200bp, the percent of G or C nucleotides must be ≥ 50%, observed/expected CpG ratio must be ≥ 0.6, and an additional running score is calculated that must remain above 0 for the entire length of the island. The running score is computed by adding 17 for each CpG, and subtracting 1 for every other base. Also, islands are cut in half at their maximum running score and each half is evaluated separately (for details see http://genomewiki.ucsc.edu/index.php/CpG_Islands).

Additionally, CpG island predictions from Wu et al. [27] were downloaded from the paper’s website if available (*Gallus gallus*, *Mus musculus*, *Homo sapiens*), or calculated from scratch using default parameters with the exception of *Xenpous tropicalis*, which repeatedly crashed and was therefore excluded from our analysis. The method uses a hidden Markov model in 16 bp steps, with the surrounding 256 bp windows of the genome as observations, and models three states: Alu repeat elements, CpG Islands, and “baseline” (the rest of the genome), with the regions belonging to the Alu state provided as known.

It should be noted that both methods use an unsupervised learning approach, meaning that they do not use a training set of genomic regions that are known to be either methylated or non-methylated. This is because they were both developed prior to the creation of experimental methods that are able to identify large sets of regions which would be suitable for this training set.

### Spectrum Kernel Support Vector Machine

As a proven supervised machine learning method we used a support vector machine with a spectrum kernel to predict non-methylated islands genome-wide. A support vector machine (SVM, [31]) finds a boundary between two sets of data: positive and negative training data. This boundary can then be used to classify new data points by checking to see which side of the boundary the data points fall on. The spectrum kernel [32] is a string kernel that is known to perform well on many biological applications [21, 33], especially those where strings of genomic sequence are the primary input. A spectrum kernel is one of the simplest string kernels, and as features it counts the frequency of k-mers (k-length subsequences) in each of the input sequences. The similarity between two sequences is then the dot product of the vectors of these frequencies.

Feature weights (*w*) for each k-mer are calculated as outlined in the original spectrum kernel paper of Leslie et al. [32]:

$$\begin{array}{c} w=\sum_{\text{support vectors }x_{i}}\alpha_{i}y_{i}\Phi \left(x_{i}\right) \end{array}$$ (1)

where *α* is the optimal solution to the SVM dual problem, *y*_*i*_ is the NMI’s class (-1 or 1), and Φ is the spectrum of k-mers for the sequence *x*_*i*_.

The spectrum kernel implementation we used is contained in the Shogun machine learning toolbox [34], and SVM solver that was used is SVMlight [35].

### Genome Preprocessing

Each genome was pre-filtered to remove regions that are not uniquely mappable using the GEM mappability program [36] (version 20130406-045632). This was done because the gold standard Bio-CAP data is sequencing-based, and therefore is blind to NMIs that are in regions that are not uniquely mappable. GEM was run with default parameters with the following exceptions, which were chosen in order to match the sequencing data and read mapping settings that were used when defining NMI peak regions [20]: generate an index that includes the reverse complement DNA sequence, use a read length of 51bp, and allow a maximum of 2 mismatches.

We additionally removed a region of 500bp centered at the borders of each NMI from the analysis. This was done because Bio-CAP is not a high resolution method, and therefore there is some uncertainty about the exact location of the NMI borders. The border regions were removed so that we could avoid training the classifier on mislabeled regions, and to hopefully reduce the number of false positive and false negative regions in our data sets overall.

### Performance Metrics

The performance of all NMI classifiers was evaluated using two metrics: the area under the receiver operating characteristic curve (AUROC), and the area under the precision-recall curve (AUPRC). Both methods require a set of regions which have been scored according to their likelihood of being an NMI or not, as well as the known NMI class of the regions.

Receiver operating characteristic curves plot the true positive rate versus the false positive rate of the classifier as the score threshold that is used to determine whether a region is an NMI or not is varied across all possible values. The area under this curve is then calculated and can be interpreted as the probability that a randomly selected NMI region will score higher than a randomly selected non-NMI region. Values near 0.5 indicate that the classifier performs similarly to randomly selecting a class, and an AUROC of 1.0 is a perfect classifier.

Precision-recall curves were also used to evaluate our classifiers. This is because ROC curves are known to be insensitive to class imbalance [37], or the ratio of NMI regions versus the rest of the genome. Because NMIs are quite rare in the genome, only making up roughly 2-4% of each organism’s genome, when trying to predict their location genome-wide we are in a situation where there is a severe class imbalance. This is a difficult situation because a classifier that appears to perform well in the balanced setting might be nearly useless in the unbalanced setting, because it is very hard to control the number of false positives in such a situation. To give a better idea of how practical these methods are for the genome-wide prediction of NMIs it is better to look at precision-recall curves, which plot the fraction of predicted NMIs that are true NMIs (precision) versus the fraction of all true NMIs that are predicted as NMIs (recall) as the score cutoff for calling a region an NMI is varied across all possible values. This gives us an idea of the false discovery rate (1—precision) of the method at different score thresholds and whether the genome-wide predictions the classifier makes are likely to contain many false positives.

### Parameter Tuning and Performance Evaluation

Parameter tuning, model training and performance evaluation was carried out separately on each organism as explained below. Two exceptions to this is in the calculation of the top 20 k-mers for each organism, and the cross-species predictions, in which case the k-mer length parameter was fixed at 6 and SVM soft margin penalty parameter C fixed to 1.0 to make the results more easily comparable across organisms.

In order to select the appropriate parameters for our SVM and to evaluate its performance when identifying NMIs, we split each organism’s filtered genome into three sets: a parameter tuning set which was used to select optimal model parameters, a separate training set and a test set to evaluate the performance of the model on held out data. A random subset of each organism’s chromosomes was set aside as the test set, such that approximately half of the organism’s genomic sequence was contained in the test set. The remaining chromosomes were then divided into NMI and non-NMI regions, and those regions were split into 750 bp windows of which a random 50% were used for parameter tuning and the other 50% were used for training. This window size was selected after considering the length distribution of the NMIs to ensure that the majority of NMIs would be longer than or equal to the window length. In order to control for the differing length of each organism’s genome as well as the different ratio of background-to-NMI sequences, the tuning and training windows were subset to have a total of 30,000 windows, with a 5:1 ratio between background and NMI sequences.

In order to identify the optimal parameters of our SVM model we chose to use a common and simple method called a grid search, which involves evaluating the SVM’s performance on a subset of the data for every possible combination of parameters using cross validation. In our case, the SVM’s soft margin penalty *C* as well as the *k*-mer length were selected in this way. A grid search was performed over *C* values of 0.01, 0.1 and 1, and *k*-mer lengths between 2 and 9. We used 5-fold cross validation on the parameter tuning data set and the parameters that yielded the highest average AUROC were selected (to see the optimal parameters for each organism and fold see S3 Table). The R package caret [38] was used for parameter tuning.

The SVM was then trained on the training set using the selected parameters, and the chromosomes in the test set were split into 750 bp windows whose NMI status was then predicted. For comparison with other methods, the windows were further split into 50 bp windows, because the predictions of the other methods are occasionally quite short (e.g. the Wu HMM method predictions have a small percentage of predictions less than 100 bp), and these predictions would be lost if the window size is too large. A window was called a true NMI if ≥ 50% of its length was covered by an experimentally determined NMI.

### Randomly Trained Model

In order to control for overfitting, as stated above we used non-overlapping parameter tuning, training and test datasets, and we choose our optimal parameters using 5-fold cross validation on the parameter tuning set. In addition, we also wanted to evaluate how our model behaves when trained on data with randomly shuffled labels. This extra check was carried out to ensure that we are not leaking information between any of the three previously mentioned sets of data, and also to evaluate the behaviour of the model when trained on a dataset with a different level of class imbalance than the final test set (5:1 in the training set versus approximately 20:1 in the test set). By using randomly shuffled class labels in our training set we are able to preserve the relative class abundance and exact number of training examples in each class.

We first looked at the results of parameter tuning (using only the parameter tuning set) when training on shuffled labels. We see that the AUROC (the criteria which we are using to choose the best parameters) when performing 5-fold cross validation on the parameter tuning set is very close to 0.50 regardless of which parameters are selected. It is noteworthy to mention that in this setting the class imbalance is the same in both the training and test set.

We then set the parameters to the same ones that were used for cross-species prediction (*k*-mer length 6, *C* = 1.0), trained on our large held out training dataset with shuffled labels and predicted on the held out test set. Unlike the parameter tuning setting described above, the class imbalance is not the same in the training and test sets. The training set has a 5:1 ratio of background:NMI sequences, because the background sequences were downsampled to keep computational runtime reasonable. In contrast the test set has an imbalance that differs based on the organism, since the test set is made up of approximately 50% of the organism’s genome, and is somewhere around 20:1 background:NMI sequences.

The results show that the AUROC is approximately 0.35-0.65 for this classifier, likely reflecting the effect of a different amount of class imbalance between the training and test set. AUPRCs are close to the expected value of approximately 0.015 which would be achieved by a classifier which randomly assigns a class. These results have been added to Figs 1 and 2 (ROC and PRC plots respectively), as well as Table 1 as “SVM (random)”.

### Cross-Species Prediction Evaluation

When performing cross-species prediction, we reused the same training and test sets that were defined for the intra-species prediction. This ensures that the performance of cross-species prediction can be more directly compared to the intra-species prediction because they both use the exact same training and test sets, and therefore preserve several properties that could effect training performance including training set size, any possible chromosomal biases, and training and test set class imbalance.

### Software Availability

We have produced a set of scripts that can be used to train a classifier and predict NMIs in a set of DNA sequences. This code is available at https://github.com/matthuska/predict-nmi, and is licensed under the MIT open source license. These scripts use the spectrum kernel SVM from the Shogun machine learning toolbox [34].

## Supporting Information

S1 Table Performance measures for all methods of NMI prediction at a single score cutoff. The Wu HMM and UCSC methods are already provided as windows, so it is not possible to compute AUROC and AUPRC curves for them. Therefore we present several other common performance measures, and apply a score cutoff to the CpGRatio and SVM methods in order to make them comparable. The SVM cutoff was chosen at a score of 0, and the CpG Ratio score cutoff was 0.6, to match the original Gardiner-Garden and Frommer method. FPR is the false positive rate and TPR is the true positive rate. The performance of the Wu HMM method for Xenopus tropicalis was intentionally left out, because the method consistently crashed when trying to calculate predictions for this genome. All values are averages across 5 random splits of the genome into parameter tuning, training and test sets. Recall is not included because it is identical to the true positive rate.
(CSV)

S2 Table Comparison of highest weighted 6-mers features from a trained string kernel SVM with known JASPAR core vertebrate motifs using the tool Tomtom. 6-mers were ranked by average feature weight across 5 random splits of the genome into parameter tuning, training and test sets. The top 20 k-mers with the highest weight (contributing most to classifying a sequence as a NMI), and bottom 20 k-mers (contributing most to classifying the sequence as a non-NMI) were used. Only motif matches with a q-value less than 0.1 were kept. The Query ID consists of the position of the k-mer in the feature weight ranking, the k-mer sequence itself, whether it contributes positively or negatively to classifying the sequence as an NMI, and the normalized feature weight.
(CSV)

S3 Table Optimal SVM parameters for each organism for 5 random splits of the genome into parameter tuning, training and test sets. In order to determine the optimal parameters for our SVM, the soft margin penalty “C” and the k-mer length, a grid search was performed and 5-fold cross validation was used to estimate the classification error on the parameter tuning set of genomic regions. This was carried out 5 times, on different random splits of the genome into parameter tuning, training, and test sets in order to determine how stable these parameters are. The optimal parameters are listed, along with the average AUROC they achieved across 5-fold cross validation. A random classifier would be expected to achieve an AUROC of 0.50.
(CSV)
